# Prospective Evaluation of Cases of Discharge against Medical Advice in Abuja, Nigeria

**DOI:** 10.1155/2015/314817

**Published:** 2015-03-02

**Authors:** Bioku Muftau Jimoh, Obalim-Chris Anthonia, Igwilo Chinwe, Adewumi Oluwafemi, Aremu Ganiyu, Adamu Haroun, Eziechila Chinwe, Aiyekomogbon Joshua

**Affiliations:** ^1^Department of Surgery, Federal Staff Medical Centre, Abuja, Nigeria; ^2^Department of Obstetrics and Gynecology, Federal Staff Medical Centre, Abuja, Nigeria; ^3^Department of Internal Medicine, Federal Staff Medical Centre, Abuja, Nigeria; ^4^Department of Pediatrics, Federal Staff Medical Centre, Abuja, Nigeria; ^5^Department of Radiology, Federal Staff Medical Centre, Abuja, Nigeria

## Abstract

*Background*. Discharge against medical advice (DAMA) is a global clinical phenomenon contributing significantly to adverse patients' outcome. Literatures abound on self-discharges in specific medical subpopulations. However, multidisciplinary studies on this subject in our region are few. *Aim*. To prospectively evaluate cases of DAMA in a wholesale multidisciplinary perspective at Federal Staff Medical Centre, Abuja, and suggest strategies to reduce it. *Patients and Methods*. All consecutive patients who DAMA from our medical centre between June 2013 and May 2014 were included in the study. Data harvested from the standard proforma were analyzed using IBM SPSS version 19.0. *Results*. We recorded an overall DAMA rate of 2.1%. The majority of the patients were paediatric cases (*n* = 63, 44.6%) while closed long bone fractures represented the leading diagnosis (*n* = 35, 24.8%). The most commonly cited reasons for leaving the hospital were financial constraints (*n* = 46, 32.6%) and seeking alternative therapy (*n* = 25, 17.7%). *Conclusion*. The DAMA rate in our study is comparable to some urban hospitals elsewhere. However, the leading reasons for this phenomenon are unacceptable in the current medical best practice. Thus, strengthening the Health Insurance Scheme, strict control of traditional medical practices, and focused health education are recommended strategies to reduce DAMA.

## 1. Introduction

Every year in our region, thousands of patients leave the hospital before the treating physicians recommend their discharge [1]. Variously abbreviated as DAMA (discharge against medical advice), SAMA (signing against medical advice), LAMA (leaving against medical advice), or DAOR (discharge against own risk), the phenomenon poses serious clinical, ethical, and legal challenge to the individual physician as well as the hospital.

Researches show that DAMA is associated with higher patients' morbidity and mortality. It could also result in readmission [2–9] and complications, longer hospital stays, and higher costs of treatment [10, 11].

There is considerable variation in the prevalence rate of DAMA, ranging from 0.7% to 2.2% [1, 2, 12, 13] among general hospital patients, but may reach up to 25.9% in other centres [5, 7, 14]. Also, some studies have documented a higher rate of DAMA in developing than developed countries [3, 15–17].

The reasons often cited by patient for DAMA are legions. In addition to financial constraints, perceived improvement in clinical state and preference for alternative therapists like traditional bone-setters were prominent in some local studies [17–19]; low levels of trust, partnership, and communications between patients and their doctors were responsible in others [20–24].

In our environment, literatures on this subject are few and mostly retrospective. They focused on patients leaving against medical advice in specific medical subpopulations such as orthopedic, psychiatric, medical and substance abuse [2, 5, 14, 19, 25–27]. Hence, the current study aims to prospectively evaluate cases of DAMA in a wholesale multidisciplinary perspective and proffers strategies for reducing this unwarranted but relatively common clinical entity [28–30].

## 2. Patients and Methods

All consecutive patients who left against medical advice from the medical, obstetric and gynaecological, paediatric, and surgical wards of Federal Staff Medical Centre, Abuja, between June 2013 and May 2014 were prospectively included in the study. Data harvested from the standard proforma, as completed by one of the authors and any doctor-on-duty, included patients' demographic variables, educational status, the relation to the patient (for paediatric cases), the diagnoses, and reason(s) for DAMA. The institution ethical committee's approval was sought. The data were subsequently analysed using IBM Statistical Package for Social Science version 19.0 for Windows. *P* value ≤ 0.05 was considered significant.

## 3. Results

We found that, of the 6,741 cases admitted, 141 patients left the hospital against medical advice, giving an overall DAMA rate of 2.1%. Within the study period, 66 males (44.6%) and 78 females (55.4%) were discharged at own risk with ages ranging from 2 hours to 85 years (mean age = 21.3 years) (Table 1).

**Figure 1:**
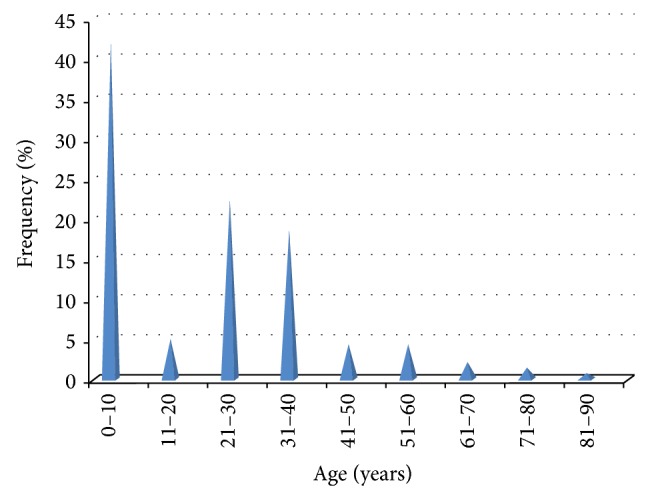
Age distribution of patients who obtained DAMA.

**Figure 2:**
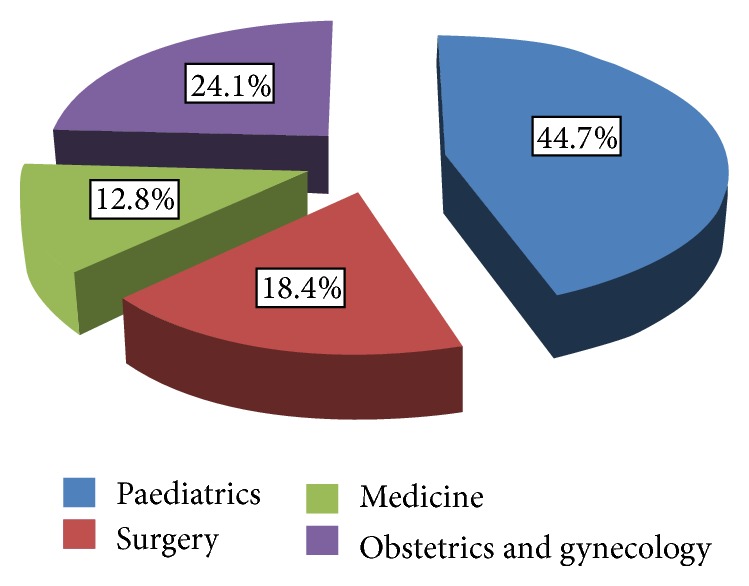
Distribution of DAMA by specialties.

Figure 1 depicts the age distribution of DAMA cases. It was noted that preponderance of patients was signed by parents or caregivers against doctor's counsel (*n* = 66, 46.8%). Departmentally, the data showed that the majority of the patients who have DAMA were paediatric cases (*n* = 63, 44.6%) while obstetric/gynecological, surgical, and internal medical patients accounted for 34 (24.1%), 26 (18.4%), and 18 (12.7%) patients, respectively (Figure 2).

In our study, closed long bone fractures represented the highest number of DAMA (*n* = 35, 24.8%). Infections, severe hypertension, severe malaria, and neonatal jaundice were diagnosed in 27 (19.1%), 20 (14.2%), 18 (12.8%), and 17 (12.0%) patients, respectively. Other diagnoses included severe dehydration secondary to acute gastroenteritis (*n* = 11, 7.8%) and complicated diabetes mellitus (*n* = 7, 5%) (Table 2).

**Figure 3:**
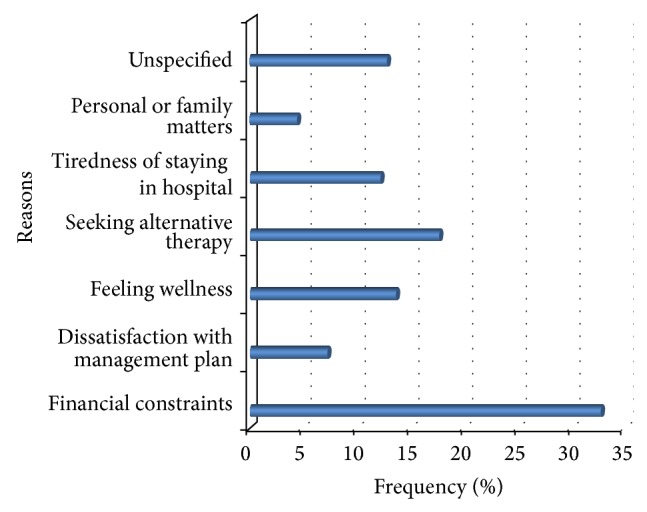
Reasons cited for discharge against medical advice.

The reasons for signing against medical advice are presented in Figure 3. The most commonly cited reasons for leaving the hospital were financial constraints (*n* = 46, 32.6%), dissatisfaction with management plan (*n* = 10, 7.1%), feeling of wellness (*n* = 19, 13.5%), seeking alternative therapy (*n* = 25, 17.7%), tiredness of staying in the hospital (*n* = 17, 12.1%), attending to personal or family matters (*n* = 6, 4.3%), and unspecified (*n* = 18, 12.8%).

## 4. Discussion

This is one of the reports with multispecialty outlook on patients who signed against medical advice in our region. We noted an overall DAMA prevalence rate of 2.1% which is comparable to that of 0.8–2.2% documented in some teaching and acute care hospitals in United States [2, 5, 25, 26]. A similar local study by Alebiosu and Raimi [17] has rate of 2.8% though accident and emergency patients (which accounted for 45.2%) of all DAMA were included in their work. However, it contrasts sharply with the finding of Eze et al. [18] who recorded a rather low prevalence rate of 0.002%. The resemblance of our picture to the former one was due to the location of the medical centre in municipal area council of the federal capital.

There was no statistically significant sex bias among our patients who have DAMA (*P* > 0.05). However, some studies [2, 4, 6, 30–32] have reported male sex, younger age, poor social support and lack of health care coverage, psychiatric illness, and substance abuse to be frequently associated with self-discharge. The male gender preponderance in local reports [28, 29, 33, 34] was mainly trauma based.

A wide range of diagnoses were recorded in self-discharge cases in our work with trauma-induced closed long bone fractures leading the pack (25%). This is corroborated by other series [29, 31, 34]. These are the categories of patients who, due to cultural influences, also patronize traditional bone-setters and thus obtained DAMA. Other diagnoses include infections, severe hypertention, severe malaria, neonatal jaundice, and severe dehydration due to acute gastroenteritis.

In this part of the world, priority is accorded to rituals of naming of newborns at the end of first week of delivery. Parents could do anything to see that these ceremonies are performed at home. Thus, child patients admitted on account of severe pathologies are discharged prematurely.

We observed that financial constraint was the commonest explanation advanced by patients to justify leaving the hospital against doctors' wish (32.9%). This was also noted in other literatures [15, 17, 34]. This could be due to widespread poverty and lack of access to National Health Insurance Scheme (NHIS) by the majority of our populace. Patients are thus left to bear the cost of treatment alone even in emergencies. The relatively high cost of orthodox health care in our centres may also contribute. Furthermore, 25% of patients who have DAMA preferred alternative therapy from traditional bonesetters or traditional birth attendance for multiple reasons including fear of surgery (amputation or cesarean section), lower cost, and possibility of resolution of their problems both physically and spiritually. However, some of these patients are mismanaged and have to be readmitted with increased morbidity and cost of treatment [6, 10, 11, 33].

Significant numbers of DAMA were dissatisfied with our management plan (7.1%), tired of staying in the hospital (12.1%), and felt well enough to go home (13.5%). These may be a result of ineffective communication between the attending doctor and patient regarding the history of the disease and its prognosis, complications, and outcomes of available treatment options [35, 36]. Given the current drive for short stay practice, most of those who obtained DAMA on the stated grounds can be discharged on specific instructions or on request. Other factors which could be responsible for patients' dissatisfaction with care are psychosocial dispositions, psychiatric diseases, and substance abuse [12, 36, 37]. However, these were not evaluated in this study.

## 5. Conclusion

The DAMA rate in our study is comparable to some urban hospitals elsewhere. However, the leading reasons for this phenomenon are unacceptable in the current medical best practice. Thus, there is need to strengthen and expand the scope of NHIS [37] while reducing the cost of treatment in our hospitals. In addition, strict legislation and control of traditional medical practices and more importantly focused health education on the potential benefits of orthodox medicine compared to alternative care are recommended strategies to reduce DAMA.

## Figures and Tables

**Table 1:** Demographic characteristics of the study population.

Characteristics	*N*
Total admission	6,741
DAMA	141
Males	63
Females	78
M : F ratio	1 : 1.2
Age range	2 hours, 85 years
Mean age	21.3 years

**Table 2:** Diagnoses of patients who have DAMA.

Diagnosis	*N*	%
Fractures	35	24.8
Infections	27	19.1
Severe hypertension	20	14.2
Severe malaria	18	12.8
Neonatal jaundice	17	12.0
Severe dehydration	11	7.8
Complicated diabetes mellitus	7	5.5
Others	6	4.2
Total	**141**	**100**
